# Microarray Analysis in the Archaeon *Halobacterium salinarum* Strain R1

**DOI:** 10.1371/journal.pone.0001064

**Published:** 2007-10-24

**Authors:** Jens Twellmeyer, Andy Wende, Jan Wolfertz, Friedhelm Pfeiffer, Markus Panhuysen, Alexander Zaigler, Jörg Soppa, Gerhard Welzl, Dieter Oesterhelt

**Affiliations:** 1 Max-Planck-Institute of Biochemistry, Membrane Biochemistry, Martinsried, Germany; 2 Max-Planck-Institute of Psychiatry, Molecular Neurogenetics, Munich, Germany; 3 Institute of Molecular Biosciences, University of Frankfurt, Frankfurt am Main, Germany; 4 Institute of Biomathematics and Biometry, Forschungszentrum für Umwelt und Gesundheit (GSF)-National Research Centre for Environment and Health, Neuherberg, Germany; University of Massachusetts, United States of America

## Abstract

**Background:**

Phototrophy of the extremely halophilic archaeon *Halobacterium salinarum* was explored for decades. The research was mainly focused on the expression of bacteriorhodopsin and its functional properties. In contrast, less is known about genome wide transcriptional changes and their impact on the physiological adaptation to phototrophy. The tool of choice to record transcriptional profiles is the DNA microarray technique. However, the technique is still rarely used for transcriptome analysis in archaea.

**Methodology/Principal Findings:**

We developed a whole-genome DNA microarray based on our sequence data of the *Hbt. salinarum* strain R1 genome. The potential of our tool is exemplified by the comparison of cells growing under aerobic and phototrophic conditions, respectively. We processed the raw fluorescence data by several stringent filtering steps and a subsequent MAANOVA analysis. The study revealed a lot of transcriptional differences between the two cell states. We found that the transcriptional changes were relatively weak, though significant. Finally, the DNA microarray data were independently verified by a real-time PCR analysis.

**Conclusion/Significance:**

This is the first DNA microarray analysis of *Hbt. salinarum* cells that were actually grown under phototrophic conditions. By comparing the transcriptomics data with current knowledge we could show that our DNA microarray tool is well applicable for transcriptome analysis in the extremely halophilic archaeon *Hbt. salinarum*. The reliability of our tool is based on both the high-quality array of DNA probes and the stringent data handling including MAANOVA analysis. Among the regulated genes more than 50% had unknown functions. This underlines the fact that haloarchaeal phototrophy is still far away from being completely understood. Hence, the data recorded in this study will be subject to future systems biology analysis.

## Introduction

Regulation of gene expression is crucial for the survival of virtually all organisms on earth. It enables cells to adapt to changing environmental conditions. By expressing certain genes only if necessary the cells save both energy and resources. Basically, in archaea the phenomenon of gene regulation is less understood. One tool to study differential gene expression is the DNA microarray technique. It was developed in the early nineties of the last century. Since then it was used in numerous studies. However, its application in the investigation of gene regulation in archaea is still very limited.

*Hbt. salinarum* has found its niche in saturated brines. Beside this specialization it has to cope with numerous environmental changes. Among them is the availability of oxygen as terminal electron acceptor and of light as alternative energy source. If oxygen is available, the cells utilize a respiration chain to maintain a proton gradient. Lowering oxygen tension and increasing illumination lead to overexpression of bacteriorhodopsin (BR) [1], [2]. BR is a light-driven proton pump which is able to maintain a proton gradient to drive ATP production via an ATP synthase. When oxygen is depleted, light can even serve as the only energy source and the cells grow phototrophically [3]. Hence, the conditions that induce the archaeal retinal based photosystem are the same as in the case of the bacterial chlorophyll based photosynthetic system. In the eighties of the last century the use of mutants was introduced to reveal a regulatory network underlying BR expression. Among the regulatory proteins Bat and Brp were identified as the oxygen sensor and the light sensor, respectively [4]. However, Peck *et al.* also provided experimental evidence that Brp may be involved in retinal synthesis, either on a regulatory or catalytic level [5]. In more recent times global systems analyses were performed using tools such as transcriptomics and proteomics [6], [7]. In these studies different *Halobacterium* spp. were compared in order to elucidate gene expression events governed by phototrophy. The authors found that synthesis of CrtB1, a key enzyme in carotenoid biosynthesis, correlates with the expression of bacterioopsin. Furthermore, they showed that ATP-producing arginine fermentation is regulated inversely to the expression of BR.

Recently, these changes were also analyzed by quantitative proteomic approaches, but these studies are still hampered by the incomplete quantitative analysis of proteins. Although tools such as ICPL have expanded the number of proteins, which can be quantified, the changes of more than 50% of the theoretical proteome is still not detectable by these approaches [8, Tebbe *et al.*, submitted]. Hence, transcriptional analysis is still superior in regard to the completeness of the analysis of regulatory events. Second, RNA analyses are capable to observe differential expression of genes, which encode cytosolic as well as membrane proteins, with comparatively low technical effort.

Here we describe a PCR-product based DNA microarray approach for examination of genome-wide transcriptional changes that also reliably detects faint changes in gene transcription. We applied our tool to analyze the transcriptional differences in a fundamental biological phenomenon that is driven by surprisingly faint changes in gene expression, i.e. phototrophy in the euryarchaeon *Halobacterium salinarum* strain R1. For that it was important to optimize the data analysis procedure in order to get reliable data of the weak expression differences. We present the first DNA microarray data set of cells actually grown under anaerobic, phototrophic conditions.

## Results

### Experiment design

To better understand the complex regulations underlying the adaptations of the energy metabolism we established a whole-genome DNA microarray for *Hbt. salinarum* strain R1. The manufacture of the microarray was facilitated by the sequencing and annotation of the genome in our group (see www.halolex.mpg.de; [9], [10]). Taking into account that aerobic and anaerobic conditions represent very different physiological growth conditions, we initially expected large changes in gene expression. However, previous proteomic results proved the differences in gene expression to be rather small [8, and Tebbe *et al.*, submitted]. In these studies, very few genes were found to be regulated more than threefold. Consequently, the challenge was to detect such small changes in transcript abundances by a high-throughput approach.

Technical as well as biological replications are of great importance for successful transcriptome experiments [11]. We analyzed five independent cultures for each growth condition. The cell cultures were carefully adapted to the respective growth condition prior to transcriptome analysis. Further, samples for RNA isolation were drawn from exponentially growing cells. Our DNA microarray contains five replicates of each gene probe. The cDNAs were hybridized applying the common reference design. For that a “common reference”-cDNA was generated from a RNA mixture that contained all RNA samples in equal amounts. This cDNA served as reference in all hybridizations [12].

### Data processing

Due to the fairly small regulation levels it was crucial that the microarray data were processed very carefully. There are numerous software packages for the analysis of spotted microarray experiments. But specific requirements related to experiments and technical conditions make it lucrative to be able to program an individual data preprocessing system using an object-oriented language and integrating existing modules. Hence, the following paragraphs describe shortly how the fluorescence data were preprocessed and statistically analyzed using the MAANOVA package [13] in the R environment (see http://www.r-project.org/).

The fluorescence values of all DNA probes derived from the ten microarrays (5 from aerobic and 5 from phototrophic cells) were collected in a text file. The data were subjected to several preprocessing steps, which included a background correction and two normalization procedures. All these steps were conducted with algorithms written for use in the R environment (source codes available upon request).

A first description of the expression values showed two problems: censored values and values similar to background values. Saturation of the fluorescent signal results in censored data. These data are a specific form of missing data and must be handled carefully.

**Figure 1:**
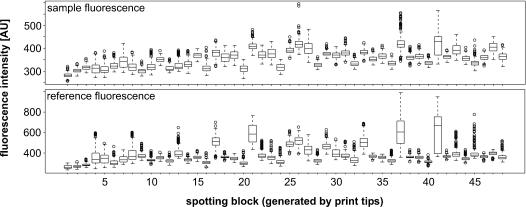
Box plots of background sample (upper diagram) and reference fluorescence (lower diagram) values around probe spots. The values are grouped according to the spotting pin. The horizontal line within the boxes shows the median. The borders of the boxes show the 75th and 25th percentiles. The dotted lines outside the boxes indicate upper and lower limit values. Open circles represent individual outlier spots.

**Figure 2:**
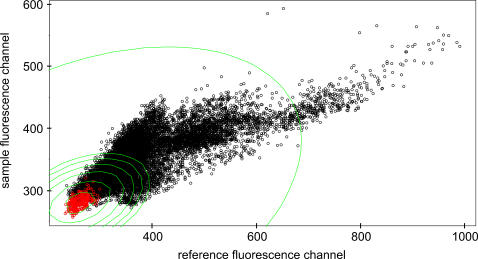
Scatter plots of local background values of probe spots in reference and sample fluorescence channel. The contour lines (green) were calculated using the minimum volume ellipsoid method for all spots printed by an individual pin (red).

Background subtraction in general intensifies the problem of generating negative or small values with extreme and unreliable ratios. In our data sets the background fluorescence varied significantly throughout each microarray, but there was a clear coincidence between the green and red fluorescence channel (Figure 1). Consequently, it was feasible to subtract the local background fluorescence from the fluorescence of each probe spot without introducing biases in expression values. Because we wanted to keep as much as possible low intensity signals, we did not simply exclude all fluorescence data below a certain threshold. We used the dependency of green and red fluorescence structure to construct a distance based on the contour lines of a two dimensional normal distribution (Figure 2). Here, it was necessary to use robust estimators for means and standard deviations (Minimum volume ellipsoid, [14]).

**Figure 3:**
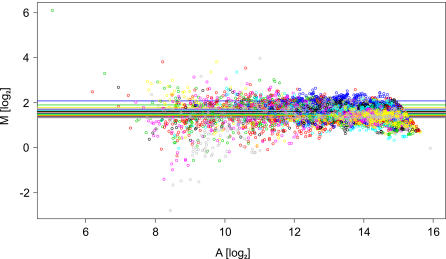
MA plot of raw fluorescence data within a single microarray. The data are colored pin-wise (fluorescence intensities obtained from probes spotted by a certain pin are colored individually).

**Figure 4:**
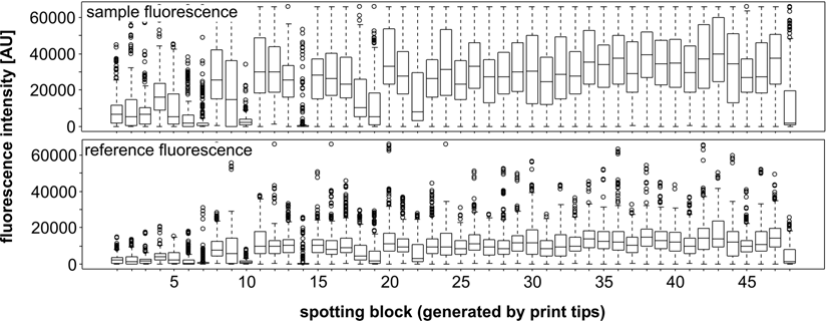
Box plots of sample (upper diagram) and reference fluorescence (lower diagram) values of probe spots. See legend of Figure 1 for a detailed description.

There are plenty of biases, which are introduced to microarray experiments for technical reasons, e.g. by printing probes with a multi-pin head, by different incorporation efficiencies of the fluorescent dyes, or by just different fluorescence intensities of individual probes [15]. All these variations have to be carefully inspected and removed before extracting any gene regulation from the data set. Among the methods available a regional median normalization was applicable for our data sets. The indications for that were (i) no dependence of the fluorescence ratios of the two dyes from the fluorescence intensity (Figure 3), (ii) a clear spatial effect generated by spotting of the microarray with a 48-pin print head (where each pin printed one of 48 blocks of probe spots) (Figure 4). Consequently, we normalized the fluorescence ratios so that the median of all probes spotted by the same pin is 1.

The aim of the statistical analysis was to identify differentially expressed genes when comparing aerobically and phototrophically grown cells. The R/*maanova* package (version 0.97-4) provides a complete work flow for microarray data analysis. Especially “mixed effect ANOVA” models are implemented to estimate variance components and to perform F- and t-tests for differential expressions.

One problem with R/*maanova* is that it does not tolerate missing, zero or negative intensity data. The R/*maanova* manual suggests to use non-background subtracted data as input and to ignore bad flags. In our opinion this is risky because on the other hand biased expression values with many unreliable quantities are used. The application of only complete data would cause a strong reduction of the data matrix. Therefore it is necessary to make use of methods which impute missing values. In our data we observed 72 genes (2.7%) with more than 33% missing values. These genes were not further analyzed. Missing values of remaining genes were imputed using an algorithm based on Principal Component Analysis [16].

With these completed data we used the R environment MAANOVA to calculate test statistics for testing the null hypothesis *no difference between aerobic and phototrophic growth condition* for each gene. The MAANOVA package provides four test statistics, i.e. F1, F2, F3, and Fs. We applied the Fs statistic, which uses a shrinkage estimator for gene-specific variance components based on the James-Stein estimator.

The application of formal hypothesis testing provides p-values. These p-values from tests of differential expression summarize the statistical significance of the applied test statistic, based on the variation in gene expression and the error variance. To cope with the problem of multiple test false discovery rates (FDR) adjusted rates were calculated using the step down method [17]. The ranking of the genes is based on these adjusted p-values.

### Expression differences between cells adapted to aerobic and phototrophic conditions

A rank number was associated to all genes, which were analyzable. The rank sorts the genes from the most statistical significantly regulated to the least significantly regulated one. We applied a false discovery rate of 5%, which resulted in a list of 239 significantly regulated genes. Under phototrophic conditions 130 genes were induced and 109 were repressed compared to aerobic growth conditions (see Supporting Information Table S1 for the complete list of regulated genes).

**Figure 5:**
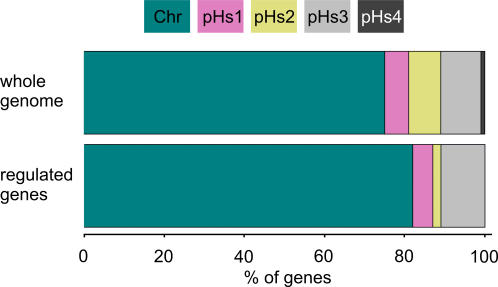
Distribution of all genes versus distribution of significantly regulated genes on the genome. Chr: chromosome; pHs1-4: plasmids.

Figure 5 shows a global inspection of the transcription differences between aerobic and phototrophic cells. It reveals that those genes, which are located on the plasmids of *Hbt. salinarum* strain R1, are obviously underrepresented. Although 25% of the genome is encoded on the four plasmids only 18% of the significantly regulated genes are located there. This difference is mainly due to the small number of regulated genes on pHs2 and no regulated genes at all on pHs4.

**Figure 6:**
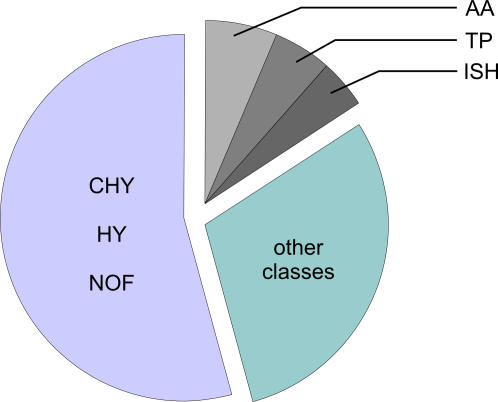
Graphical overview of the functional classification of the significantly regulated genes.

Figure 6 illustrates that more than 50% of the regulated genes belong to the function classes *hypothetical genes* (HY), *conserved hypothetical genes* (CHY), or genes with *no assigned function* (NOF). This is nearly the proportion of these three categories on the genome of *Hbt. salinarum* (60%). The huge number of 129 regulated genes without functional data illustrates, that the change of *Hbt. salinarum* strain R1 cells between aerobic and phototrophic lifestyle is less understood than one would expect. However, it should be mentioned that even *E. coli*, the best studied prokaryote on earth, has a comparably high number of genes without an assigned function.

Among the significantly upregulated genes we found *bop*, *bat*, *crtB1*, and *crtB2*. The induction of the expression of bacterioopsin, the gene product of *bop*, by phototrophic conditions was already described several decades ago [1]. Bat is a central regulatory protein necessary to activate the transcription of *bop*, whereas *crtB1* and *crtB2* encode enzymes that catalyze key steps in the synthesis of retinal [7], [18]. The transcription of all these genes enables *Hbt. salinarum* strain R1 to prosper under phototrophic conditions. Taken together, they are responsible for the maintenance of the membrane potential and proton gradient, which allows ATP synthesis without the need for a respiratory chain. Consequently, the fact that we found these genes regulated underlines that the regulation data retrieved by our DNA microarray platform are biologically relevant.

Fifteen regulated genes belong to the function class *amino acid metabolism* (AA). Seven showed increased and eight showed decreased transcript levels during phototrophic growth. Four of these genes are of particular interest: *carA*, *carB*, *arcB*, and *arcC*. The induced genes *carA* and *carB* encode a carbamoylphosphate (Carb-P) synthase, whereas the repressed genes *arcB* and *arcC* encode proteins of the arginine deiminase (ADI) pathway. CarA/B synthesize Carb-P by consumption of 2 ATP. The purpose of the ADI pathway is to hydrolyze Carb-P for generation of 1 ATP. Obviously, contrary regulation of the two gene clusters makes sense. Because ATP is plenty in light, additional ATP generating reactions can be shut down. Synthesis of Carb-P, however, facilitates nucleotide synthesis, which in turn may enhance cell growth. Interestingly, the gene *pyrI*, encoding a subunit of the aspartate carbamoyltransferase, was also found to be induced in our study. This enzyme catalyzes the conversion of Carb-P to carbamoylaspartate, the first step in pyrimidine synthesis.

Furthermore, we found several genes involved in the energy metabolism of *Hbt. salinarum* to be regulated. Among them *nuoC*, *nuoD*, *nuoK*, *petB*, and *dmsB* showed increased mRNA levels under phototrophic growth conditions. NuoC, D, and K are subunits of the NADH dehydrogenase. PetB is part of the cytochrome bc complex. DmsB is a chain of the dimethylsulfoxide (DMSO) reductase, which utilizes DMSO as alternative terminal electron acceptor [19]. In contrast, *cydA1*, a subunit of one of the three terminal oxidases, was found to be repressed under phototrophic conditions. The genes *korA, korB*, and *acn* encode enzymes that are involved in the TCA cycle. The transcription of these genes was found to be induced under anaerobic conditions. KorA and KorB constitute the 4Fe-4S protein α-ketoglutarate-ferredoxin oxidoreductase, which enables an organism to assimilate CO_2_ by a reductive TCA cycle. Acn is the aconitate hydratase that catalyzes the conversion of citrate to isocitrate.

Its chemotactic apparatus enables *Hbt. salinarum* to move through its aquatic habitat in order to keep itself at favorable conditions. This very important feature is controlled by a set of 18 sensor proteins, the so-called halobacterial transducers (Htrs), and the bacterial-like CheAY-system [20], [21]. Under phototrophic conditions we found a repression of 4 Htr encoding genes, i.e. *htrI*, *htrVIII*, *basT*, and *htr18*. HtrI is involved in phototaxis dependent on orange and UV light, HtrVIII is responsible for aerotaxis, and BasT enables *Hbt. salinarum* to sense the presence of the amino acids Leu, Ile, Val, Met, and Cys [22], [23], [24]. Finally, for HtrVIII no functional data exist. At least the repression of HtrI and HtrVIII can be easily explained. Typical for phototrophic conditions are strong light intensity and lack of oxygen. These circumstances obviously eliminate the need for high numbers of light sensors and for the presence of oxygen sensors. Additionally to the chemotaxis system, gas vesicles can provide a unidirectional movement of cells, i.e. floating to the surface of a water body. Interestingly, *Hbt. salinarum* induced as well as repressed the expression of gas vesicle genes. The genes *gvpL1*, *gvpK1*, and *gvpI1* on plasmid pHs1 were induced under phototrophic conditions, whereas *gvpI2* and *gvpC2* on the chromosome were repressed. However, the interpretation of that phenotype is not trivial, since gas vesicle formation in *Hbt. salinarum* R1 is generally impaired by a transposon element in front of the plasmid-borne *gvp* operon [25], [26].

Four genes of the function class *transport* (TP) were induced and 8 genes were repressed under phototrophic conditions. Most of them encode ABC transport proteins such as permeases, substrate-, and ATP-binding proteins. Basically, the action of ABC transporters should be aided by the abundance of ATP in light. The induction of 4 genes may be explained by the need for the substrates, which are imported by these ABC transporters. Accordingly, repression of 8 transport genes may point to the fact that these are specific to substrates, which are not necessary for phototrophic growth. The general problem with the interpretation of transport protein regulation is that the substrate specificity is not accessible via homology search.

Finally, 11 transposases (function class ISH) showed increased transcript levels under phototrophic conditions. The proteins belong to the COGs 0675, 1943, and 3385. We can just speculate about the reason for the activation of these mobile elements. It might reflect a certain flexibility of the genome. Generally, *Hbt. salinarum* seems to be very sensitive to environmental changes, because e.g. even a simple freeze-thaw cycle induces mutations that result in phenotypic differences (D Oesterhelt, unpublished observation).

### Validation of transcriptional regulations via quantitative RT-PCR

Due to the small regulation levels it was essential to validate the transcriptional differences found in the DNA microarray experiment by an independent method. For that we chose a quantitative two-step reverse transcription-PCR (RT-PCR). In the first step total RNA was unspecifically transcribed into cDNA by using random hexamer primers. In a second step quantitative real-time PCR was applied using the gene-specific primers shown in Table 1. For that, 6 of the 10 RNA samples (3 of 5 from each growth condition) used for the DNA microarray experiment were applied to RT-PCR. In order to get an overview of the reliability of the microarray data we performed RT-PCRs of 17 genes. Ten of which having statistical ranks from 4 to 221 and, hence, belong to the group of significantly regulated genes. The other 7 genes are distributed over the range of statistically insignificant genes (having statistical ranks from 332 to 2411).

**Table 1:** Sequences of primers used for quantitative RT-PCR analysis.

Gene ID	Forward primer (5′–3′)	Reverse primer (5′–3′)
OE1710R	AACCCGGACCTCGAAGTGAT	CCGTAGATGCGGTTGTCCAT
OE2225F	AGAACAACGTCCCGATGGG	TCGGCTGGTAGGTCATTTCG
OE2868R	GTTCACCCACATCTCGGTCC	GGCCAGACTCTCCAATCCAC
OE3093R	GAGGAGGTCACCACGTTCATC	GACGACCCACGGAGGTACTC
OE3100F	TCGGCACTGTCGAAGACATC	CGGAGAGCCGGTACGTGAT
OE3102R	GGCGTCTACCTGTTGGGTATG	GCAGGTTCTCGAAATGCTCG
OE3107F	TTTCCAGCGTCGCTCAACA	CAAAACGTCATGCCGAACG
OE3136F	CCGATGAATGTGATCTCCCGT	TGTACTCGCTGACGTGTTTCCC
OE3381R	TGGAGAAAACCGACCAGATCG	TTGTCCTTGAAGAAGATGCGG
OE3468R	ACTTCTACGAGCTCACCCGC	AGGACTCGAACAGCTCGTGG
OE3554F	GGTGGCCAAACGACAAGTTC	TCAGCAGGCTCTCCTCGAAG
OE3556R	GCGACACCGAGAAAATGGACT	ATAGCCGTGGTTCTGTGTGGTC
OE3980R	GCCGAAACCCTCCTGTTGT	CGAGTGCCAGACGCAGAAG
OE3983R	CTCACGTTCCTGGCTGTCG	TCGTGTATGACAGCGCCAAC
OE4217R	ATGGACATGCAGCAGATCCTC	TGGCGTTGTAGACGATCTTGAC
OE5205R	TTCGAGCACGACGAGATGATG	ATCGTGAACATGTCGGTGAGC
OE5206R	TCGACATCGTTGAGGCAGAA	AGCACGATCCTTGTCGATGAC
OE5208R	AGGCCATCACGAACTTCCTGA	ATCGTAGTCCTCCTGGATCCCA
OE7065F	ACGTACGGACTAACGAGCAGC	GGAAGCTCATTGGGATACCG

The 10 significantly regulated genes showed a differential expression in the RT-PCR as well (Table 2). But only in the case of OE3107F and OE3136F the expression ratio was quite high (>10). Comparatively small regulation levels were also observed in quantitative proteomic studies on aerobic/phototrophic cells [8, Tebbe *et al.*, submitted]. As expected the RT-PCR data of the 7 genes with ranks lower than 239 showed much less consistency with the DNA microarray data. In the case of *arcA* and *crtI1* the RT-PCR data even indicated a gene regulation although the DNA microarray approach did not. This phenomenon is discussed in detail below. In spite of that, the RT-PCR results basically suggest that our DNA microarray platform reliably detects gene regulations, even those with small amplitudes.

**Table 2:** Comparison of microarray data and quantitative RT-PCR analysis.

Rank	ORF	Gene	Ratio MA	Ratio RT-PCR
4	OE3093R	*crtB1*	1.55	2.25
10	OE3107F		1.74	27.92
12	OE3556R	*carA*	1.64	2.23
14	OE3554F	*carB*	1.25	4.70
31	OE5206R	*arcC*	0.83	0.17
60	OE1710R	*korB*	1.27	3.17
87	OE5205R	*arcB*	0.88	0.47
139	OE2225F	*dmsB*	1.33	1.81
198	OE7065F	*cydA1*	0.78	0.67
221	OE3136F		0.75	0.07
332	OE3468R	*crtI2*	1.17	0.98
388	OE2868R	*sdhC*	0.79	0.88
500	OE3983R	*crtY*	0.86	1.36
1030	OE5208R	*arcA*	1.09	0.25
1273	OE3102R	*brp*	1.02	1.92
2290	OE3381R	*crtI1*	1.07	0.21
2411	OE3980R	*blh*	1.00	1.84

The rank is equal to the statistical significance. MA: microarray.

## Discussion

The objective of this study was to provide a tool for the analysis of regulatory events in *Halobacterium salinarum* strain R1. For that, we constructed a microarray containing probes for 97% of the genome of this halophilic archaeon. Due to the fact that we spotted every probe in five replicates the DNA microarray comprised more than 13500 probe spots. This sophisticated hardware was combined with several stringent data preprocessing steps and a statistical analysis based on the MAANOVA package of Wu *et al.* [13]. Together, our setup allows a reliable detection of gene regulations even if these are rather small. Consequently, our DNA microarray is currently applied by several further researchers to answer biological questions in *Hbt. salinarum*.

An evidence for the reliability of our data is the fact that several genes necessary for BR production were found to be induced. This phenotype is known for decades and, thus, is a good indicator for true biological relevance. Another measure for reliability is the independent verification of DNA microarray data by quantitative RT-PCR. We found that all significant genes tested by this method showed the same regulation as found by the DNA microarray analysis. Interestingly, in the RT-PCR approach nearly all genes showed higher regulation levels. This behavior is already known and led to the conclusion that microarray experiments are superior in order to identify regulatory trends in gene expression rather than giving the researcher a clue of quantitative regulation values for single genes [27].

Seven other genes were tested by RT-PCR, which were rejected by the statistical analysis as being not significantly regulated. Indeed, the coincidence between DNA microarray and RT-PCR results was clearly lower. The RT-PCR further revealed a four- to fivefold regulation for 2 genes, i.e. *crtI1* and *arcA*. However, also these two were not selected as being regulated by the microarray approach. The contradiction between DNA microarray and RT-PCR results may have several reasons: (i) cross-hybridization on the DNA microarray, (ii) only 3 of 5 RNAs from each growth condition were used for the RT-PCR analysis, and (iii) the RT-PCR data showed higher standard deviations. In case of *crtI1* cross-hybridization may be indeed the problem. The gene is highly homologous to *crtI2*, which is also among the non-significant genes, and showed no regulation in the DNA microarray as well as in the RT-PCR experiment. Though, the contradicting results for *arcA* may indicate that our stringent data handling possibly generated a reasonable number of “false negatives” among the non-significant genes. This is because *arcA* is part of the same operon as *arcB* and *arcC*, which were found to be regulated in both experimental approaches. ArcR, the putative regulator of these genes, is also encoded in this operon. According to our data *arcR* is not regulated during phototrophic growth. This corresponds well with the observation that the *arcR* mRNA is not subject to regulation but remains at a constant level no matter if aerobic or anaerobic conditions are present [28].

Transcriptome analyses enable the researcher to detect changes in expression of both cytosolic and membrane proteins. In the present study 181 of the regulated genes encode cytosolic proteins, and 53 genes encode putative integral membrane or membrane-attached proteins. Furthermore, 5 genes encode proteins, which are probably secreted into the medium. Such comprehensive description of gene expression is an advantage of microarray analyses. In contrast, proteomic analyses would need an extensive effort of time and material to give a similarly complete picture. So far, two studies reported gene expression data of *Hbt. salinarum* grown aerobically and phototrophically. Both applied quantitative proteomics tools [8, Tebbe *et al.*, submitted]. Bisle *et al.* investigated the membrane-located proteome. The investigation was still hampered by the low number of quantifiable proteins. Tebbe *et al.* applied the ICPL technique to quantify changes in the cytosolic proteome. In both studies the intensity of gene regulation turned out to be very faint. The same was found in the present analysis of gene transcript levels. As done in the present report, both proteomic studies analyzed cells, which were fully adapted to the growth conditions of interest. However, experiments conducted in our group indicate that the gene expression differences are much larger when observing the transition of cells from aerobic to phototrophic growth conditions in a time-resolved manner (J Twellmeyer, unpublished results).

Both the transcriptomics as well as the proteomics approaches showed an induction of the structural gene for bacterioopsin (*bop*) and its regulator (*bat*). Furthermore, both showed an induction of the DMSO reductase complex and a cytochrom C reductase under anaerobic conditions. These regulations, although rather weak, make sense in a physiological view and were also reported in a previous transcriptomics study [19]. Beside the regulatory overlap we also found six cases, in which transcriptomics data contradicted the proteomics results, e.g. the thiamin biosynthesis protein ThiC, the transducer protein HtrI, and a secreted hydrolase. In these cases our study showed an inverse regulation compared to the proteomics results for functionally linked proteins or for members of a polycistronic operon. Up to date it was not shown for archaea that higher transcript levels lead to higher protein levels, which is a matter of fact in bacteria. Hence, the contradictory regulations point to a partial independence of gene translation from transcriptional regulation. In fact, a recent study in *Halobacterium* NRC-1 showed that changes in mRNA abundance very often correlate with changes in protein abundance [29]. Whitehead *et al.* could also show that decreases in mRNA correlate better with decreases in protein abundance if a time lag of 30 minutes is considered. These findings further underline that transcriptomics approaches described represent a useful way to investigate gene expression changes in *Hbt. salinarum*.

So far, one transcriptomics study exists, which aimed to explore the differences in gene expression concerning energy transduction under aerobic and phototrophic conditions, respectively [7]. However, the authors applied a strategy completely different to the present study. They compared the transcriptomes of two closely related *Halobacterium* strains, i.e. NRC-1 and S9 plus two mutants descending from S9. All were grown aerobically in light. In strain S9 expression of BR is not physiologically controlled but rather it is constitutively activated. Hence, this strain was considered to mimic the anaerobic, illuminated state of *Halobacterium* cells. In contrast, we compared cells of one strain (R1) grown under aerobic (in darkness) and anaerobic (in light) conditions.

Despite of the completely different approaches a reasonable overlap of gene regulations found by each study is visible. Interestingly, the overlap is not restricted to genes relevant for BR production and assembly. Both studies revealed an unexpected high proportion of genes with unknown functions among the regulated genes. Further correlations are the repression of genes driving ATP generation by arginine degradation, and the induction of the α-ketoglutarate-ferredoxin oxidoreductase KorAB, what supports a potential reductive TCA cycle. Contradicting results concern the expression of carbamoylphosphate synthase and aspartate carbamoyltransferase. Both catalyze the synthesis of Carb-P and channel it into the nucleotide synthesis. The present study showed repression of 4 Htrs and induction of 11 transposon elements. Both of which were not observed by Baliga *et al*. On the other hand, we did not observe induction of the superoxide dismutase genes *sod1* and *sod2* by phototrophic conditions. In our view the mentioned differences illustrate the advantage of comparing cells actually growing under the conditions of interest instead of using different genotypes. Our strategy provided a broader and more complete view of the transcriptome governed by phototrophy. Especially the induction of *sod* genes is unlikely under anaerobic conditions.

Since most of the expression differences were quite faint, it is uncertain, what impact these may have on the adaptation of the organism to environmental changes. Future functional interpretation of the regulatory data will benefit from the reconstruction of the genome scale metabolic network of *Hbt. salinarum*. This *in silico* network successfully predicted already the flux of 15 amino acids during growth of *Hbt. salinarum* (Gonzalez *et al.*, submitted). Currently, the network is used to examine, which metabolic pathways are probably utilized differently under aerobic and phototrophic growth conditions, respectively (Gonzalez *et al.*, in preparation).

Finally, more than 50% of the genes found to be differentially transcribed miss any functional data or even hints to their functional role. This shows that long standing research about biosynthesis of the purple membrane, which is essential for phototrophy of Haloarchaea, has not revealed all phenomena and metabolic effects driven by this growth condition. Basically, the reliable identification of regulated genes by a high-throughput method such as the DNA microarray technique provides a solid base for further experimental analysis. However, the high proportion of unknown genes is a drawback inherent to virtually any organism. It clearly impairs the interpretation of the collected data. Combining high-throughput data sets with systems biology approaches might provide an additional tool to understand the physiology of phototrophy.

## Materials and Methods

### Growth of *Hbt. salinarum*

For aerobic growth *Halobacterium salinarum* strain R1 (DSM671) was shaken with 105 rpm in the dark at 40°C in 35 ml of complex medium [30] in 100 ml flasks. For anaerobic phototrophic growth the flasks were sealed with a silicon septum and shaken at 40°C with 105 rpm in front of a white light source in the same volume of complex medium. Cell growth was assessed by measuring optical densities using a Klett-Summerson Photoelectric Colorimeter (Klett Manufacturing, New York, USA). Cells used for RNA isolation were subcultured three times to the mid-exponential phase (cell density 30-40 Klett) under aerobic or phototrophic conditions to allow a full adaptation to the corresponding growth condition. All experiments were replicated five times with independently grown cultures.

### Manufacture of DNA microarrays

DNA probes for 2774 open reading frames (see http://www.halolex.mpg.de) of the *Hbt. salinarum* strain R1 genome were amplified by PCR. The genome-scale primer design was accomplished with the program PrimeArray [31]. The primers were purchased from Metabion (Martinsried, Germany). The sequences of the primers are available upon request. Gene amplification was carried out in 100 µl reactions with HotStarTaq (Qiagen, Hilden, Germany) according to the instructions of the manufacturer. The reaction mixture contained 20 ng genomic DNA and 0.5 µM of each specific primer. All PCR products were quality checked by agarose gel electrophoresis. Furthermore, a randomly selected subset of 20% was checked by sequencing. Sequences were verified by BLAST search against the HaloLex database (http://www.halolex.mpg.de). Finally, the DNA probes cover 97% of the *Hbt. salinarum* strain R1 genes. The PCR products were purified using MultiScreen-PCR 96-well filter plates (Millipore, Eschborn, Germany), resuspended in a 3xSSC-buffer (450 mM NaCl, 45 mM sodium citrate) containing 1.5 M betaine (*N,N,N*-trimethylglycine), and transferred to 384-well plates for arraying.

Five replicates of each probe were spotted with a 200 µm center-to-center spacing with 48 quill-pins on a Virtek ChipWriter Pro (Bio-Rad Laboratories GmbH, Munich, Germany) onto γ-amino-silane coated CMT-GAPS-II glass slides (Corning, Schipol-Rijk, NE). Subsequently, the slides were processed as described by Diehl *et al.* [32].

### Isolation of total RNA

Total RNA was extracted from cultures grown to 30-40 Klett using peqGOLD RNApure (peqLAB Biotechnology, Erlangen, Germany) according to the manufacturers instructions. After dissolving the RNA in RNase-free H_2_O the residual DNA was treated with the DNase kit DNA-free (Ambion, Huntington, UK) following the manufacturers instructions. The yield and purity of the RNA was determined with RNA 6000 Nano LabChip Kits using an Agilent 2100 Bioanalyzer (Agilent Technologies, Karlsruhe, Germany).

### Preparation and hybridization of cDNA

Synthesis of Cy5/Cy3-labeled cDNA from 5 µg DNA-free total RNA per labeled cDNA was performed using CyScribe First-Strand cDNA Synthesis Kit (GE Healthcare, Freiburg, Germany) with enclosed random nonamer primers and Cy3- and Cy5-dUTP (GE Healthcare, Freiburg, Germany) following the manufacturers instructions. Subsequently, the reaction was stopped, the RNA template was chemically degraded and the cDNA was cleaned and concentrated as described elsewhere [33]. The microarray hybridizations were designed as common reference experiments. Therefore, a pool of all RNA samples of each experiment was reverse transcribed to Cy5-labeled cDNA and served as reference in all hybridizations. Consequently, the individual RNA samples ended up as Cy3-labeled cDNA. This experiment design eliminates the need for a “dye-swap” and is more open to other experimental objectives such as transcriptome analyses over a certain time-course.

A detailed description of the hybridization procedure is given in [33]. Briefly, the microarrays were prehybridized, the Cy5- and Cy3-labeled cDNAs were mixed with hybridization buffer (30 µl final volume), applied to the microarray, and covered with a Hybri-slip HS (Sigma-Aldrich, Taufkirchen, Germany). Hybridization of the cDNA was carried out over night at 64°C in sealed hybridization chambers (Corning, Schipol-Rijk, NE). The microarray slides were washed four times and dried by centrifugation for 5 min at 600 g. The fluorescence intensities were detected using the GenePix 4000B microarray scanner and the corresponding image-analysis software GenePixPro 4.0 (Axon Instruments, Union City, USA). These raw data were further processed as described in detail in the results section. The source codes of the algorithms for preprocessing and statistical analysis of the microarray data are available upon request. The microarray data have been submitted to ArrayExpress (www.ebi.ac.uk/arrayexpress) under the accession number E-MEXP-1096.

### Reverse transcription of RNA and quantitative PCR

5 µg total RNA were reverse transcribed with 0.55 µg random hexamer primer (Promega, Mannheim, Germany) using Superscript III (Invitrogen, Karlsruhe, Germany). The quantitative PCR reactions were done in a GeneAmp 5700 Sequence Detection System (Applied Biosystems, Darmstadt, Germany) using the SYBR Green PCR Master Mix Kit (Applied Biosystems). The final reaction volume was 25 µl with 1 µl of the reverse transcription reaction as template. Primers were designed with Primer Express 2.0 (Applied Biosystems) and were applied in a final concentration of 0.2 µM (see Table 1 for primer sequences). Transcript level differences were calculated by a relative quantification approach using an internal standard gene [34]. The gene *fdx* (OE4217R) was used as internal standard, since it was shown to be constitutively expressed [35]. For all calculations the mean-C_t_ of 3 replicate reactions per primer pair was used.

## Supporting Information

Table S1 Complete list of significantly regulated genes. cytopl: cytoplasmic protein; transm: integral membrane protein; anchN_lip: membrane attached protein.
(0.17 MB PDF)
