# Supplementary material for: Microarray Analysis in the Archaeon Halobacterium salinarum Strain R1
Source: PLoS One. 2007 Oct 24;2(10):e1064. doi: 10.1371/journal.pone.0001064 (PMC2020435; doi:10.1371/journal.pone.0001064)
Supplement: Table S1 — Complete list of significantly regulated genes. cytopl: cytoplasmic protein; transm: integral membrane protein; anchN_lip: membrane attached protein. (0.17 MB PDF) [file pone.0001064.s001.pdf]

Supporting Information Table S1: Complete list of significantly regulated genes. cytopl: cytoplasmic protein; transm: integral membrane protein; anchN_lip: membrane attached protein.

Rank	ORF	Gene	Regulation (fold)	Function class[1]	Localization	Function of encoded protein
1	OE3822R		2.372	CHY	cytopl	conserved hypothetical protein
2	OE3106F	*bop*	2.089	EM	transm	bacteriorhodopsin precursor
3	OE5134F		1.558	NOF	transm	conserved hypothetical protein
4	OE3093R	*crtB1*	1.552	LIP	cytopl	geranylgeranyl-diphosphate geranylgeranyltransferase (EC 2.5.1.32) (phytoene synthase)
5	OE6063F	*lctP*	1.272	TP	transm	probable L-lactate permease
6	OE4132R	*trpS2*	1.251	TL	cytopl	tryptophan--tRNA ligase (EC 6.1.1.2)
7	OE3116F		1.312	NOF	transm	conserved hypothetical protein
8	OE5276F		-1.355	NOF	transm	conserved hypothetical protein
9	OE2780F		-3.401	HY	cytopl	hypothetical protein
10	OE3107F		1.595	CHY	transm	conserved hypothetical protein
11	OE3101R	*bat*	1.387	MIS	cytopl	bacterioopsin activator
12	OE3556R	*carA*	1.552	AA	cytopl	carbamoyl-phosphate synthase (EC 6.3.-.-) small subunit ((ammonia) (EC 6.3.4.16) or (glutamine-hydrolyzing) (EC 6.3.5.5))
13	OE5102R		1.349	ISH	cytopl	IS1341-type transposase (ISH12)
14	OE3554F	*carB*	1.247	AA	cytopl	carbamoyl-phosphate synthase (EC 6.3.-.-) large subunit ((ammonia) (EC6.3.4.16) or (glutamine-hydrolyzing) (EC 6.3.5.5))
15	OE1019R		1.379	ISH	cytopl	IS1341-type transposase (TCE32)
16	OE4121R	*ocd1*	1.273	AA	cytopl	probable ornithine cyclodeaminase (EC 4.3.1.12)
17	OE4500R	*acd6*	-1.321	LIP	cytopl	probable acyl/butyryl-CoA dehydrogenase (EC 1.3.99.-)
18	OE2778R		-1.724	HY	cytopl	hypothetical protein
19	OE1202F	*trp1*	-1.314	TP	transm	probable ABC-type transport system ATP-binding/permease protein
20	OE2779F	*pheP*	-2.070	TP	transm	amino acid transport protein (probable phenylalanine transport protein)
21	OE3324R		1.181	CHY	cytopl	conserved hypothetical protein
22	OE3842R		1.108	HY	cytopl	hypothetical protein
23	OE3930R		1.194	CHY	cytopl	protein synthesis inhibitor homolog
24	OE4668R		-1.357	NOF	transm	conserved hypothetical protein
25	OE3314R		1.454	HY	transm	hypothetical protein
26	OE4651F		1.448	COM	cytopl	probable ribose-1,5-bisphosphate isomerase
27	OE1440F		1.222	ISH	cytopl	IS1341-type transposase (TCE31)
28	OE1455R		-1.621	NOF	cytopl	conserved hypothetical protein
29	OE5248F		-1.261	NOF	transm	conserved hypothetical protein
30	OE1119F		1.233	CHY	cytopl	dTDPglucose 4,6-dehydratase C-terminal region homolog
31	OE5206R	*arcC*	-1.235	AA	cytopl	carbamate kinase (EC 2.7.2.2)
32	OE5417R		1.193	CHY	cytopl	conserved hypothetical protein
33	OE3270R		-1.340	NOF	anchN_lip	conserved hypothetical protein
34	OE5062R		1.220	ISH	cytopl	IS1341-type transposase (TCE31)
35	OE1405R		1.373	NOF	cytopl	conserved hypothetical protein
36	OE3959R	*ilvE*	1.232	AA	cytopl	branched-chain-amino-acid transaminase (EC 2.6.1.42)
37	OE4748F		-1.439	NOF	transm	conserved hypothetical protein

Rank	ORF	Gene	Regulation (fold)	Function class[1]	Localization	Function of encoded protein
38	OE2186R	*tatA*	1.280	SEC	cytopl	sec-independent protein translocase component tatA
39	OE7023R	*gvpL1*	1.391	MIS	cytopl	gas-vesicle operon protein gvpL1
40	OE1420F		-1.129	TP	transm	probable ABC-type transport system permease protein
41	OE3030R		-1.193	NOF	cytopl	conserved hypothetical protein
42	OE3057F		1.181	CHY	cytopl	conserved hypothetical protein
43	OE4382R		1.415	HY	transm	hypothetical protein
44	OE2057F	*thiC*	1.628	COM	cytopl	thiamin biosynthesis protein thiC
45	OE5110F		1.185	CHY	cytopl	conserved hypothetical protein
46	OE4336R	*nosY*	1.323	TP	transm	probable ABC-type transport system permease protein
47	OE3186F	*birA1*	-1.112	COM	cytopl	biotin--[acetyl-CoA-carboxylase] ligase (EC 6.3.4.15) 1
48	OE5039R		1.142	CHY	cytopl	conserved hypothetical protein
49	OE2170R	*tmpC*	-1.232	MIS	anchN_lip	probable periplasmic substrate-binding protein (membrane lipoprotein tmpC homolog)
50	OE5158F		1.066	HY	cytopl	hypothetical protein
51	OE1140R	*moeB*	-1.140	COM	cytopl	molybdenum cofactor biosynthesis protein moeB
52	OE1092F		-1.080	HY	cytopl	hypothetical protein
53	OE2808F		1.167	HY	cytopl	hypothetical protein
54	OE3486R	*speB*	1.118	AA	cytopl	agmatinase (EC 3.5.3.11)
55	OE5282R		1.204	CHY	cytopl	conserved hypothetical protein
56	OE2619F	*aspB3*	-1.372	AA	cytopl	probable aspartate aminotransferase (EC 2.6.1.1)
57	OE7210R		1.265	CHY	cytopl	conserved hypothetical protein
58	OE1259R		-1.203	NOF	cytopl	conserved hypothetical protein
59	OE1679R	*phoX2*	-1.208	TP	anchN_lip	probable ABC-type phosphate transport system periplasmic phosphate-binding protein
60	OE1710R	*korB*	1.268	CIM	cytopl	oxoglutarate--ferredoxin oxidoreductase (EC 1.2.7.3) beta subunit
61	OE4703R	*uae*	1.216	CHM	cytopl	UDP-N-acetylglucosamine 2-epimerase (EC 5.1.3.14)
62	OE5063R		1.319	ISH	cytopl	probable IS200-type transposase (TCE31)
63	OE2909F		-1.120	CHY	cytopl	conserved hypothetical protein
64	OE3955F	*putA*	-1.115	AA	cytopl	probable proline dehydrogenase (EC 1.5.99.8)
65	OE2973F		-1.770	NOF	cytopl	conserved hypothetical protein
66	OE3167F	*htrVIII*	-1.164	SIG	transm	transducer protein htrVIII
67	OE3762R	*glpK*	1.291	CIM	cytopl	glycerol kinase (EC 2.7.1.30)
68	OE2222F		1.455	HY	cytopl	hypothetical protein
69	OE3506F		-1.307	CHY	transm	conserved hypothetical protein
70	OE1063R		1.214	HY	cytopl	hypothetical protein
71	OE4376R		-1.161	CHY	cytopl	conserved hypothetical protein
72	OE3347F-2	*htrI*	-1.149	SIG	cytopl	transducer protein htrI
73	OE2616F		1.162	HY	cytopl	hypothetical protein
74	OE1963F	*nuoK*	1.110	EM	transm	NADH dehydrogenase (ubiquinone) (EC 1.6.5.3) subunit K
75	OE1539F		-1.217	NOF	transm	conserved hypothetical protein
76	OE5407F		1.435	ISH	cytopl	probable transposase (ISH8/ISH26)
77	OE5101R	*cat2*	-1.179	TP	transm	probable cationic amino acid transport protein

Rank	ORF	Gene	Regulation (fold)	Function class[1]	Localization	Function of encoded protein
78	OE2160R		1.415	HY	cytopl	hypothetical protein
79	OE2161R		1.136	CHY	cytopl	conserved hypothetical protein
80	OE2573F		-1.104	HY	cytopl	hypothetical protein
81	OE1124R		1.113	CHY	cytopl	conserved hypothetical protein
82	OE5339R-2		-1.188	ISH	cytopl	probable transposase (ISH1)
83	OE3717F	*nolA*	-1.280	MIS	cytopl	NADH dehydrogenase (ubiquinone) (EC 1.6.5.3) 32K subunit
84	OE1040R		1.263	ISH	cytopl	IS1341-type transposase (TCE32)
85	OE1073F		1.229	ISH	cytopl	probable IS200-type transposase (ISH12)
86	OE4221R		1.106	HY	cytopl	hypothetical protein
87	OE5205R-2	*arcB*	-1.126	AA	cytopl	ornithine carbamoyltransferase (EC 2.1.3.3), catabolic
88	OE1781F		-1.271	TP	cytopl	probable ABC-type transport system ATP-binding protein
89	OE4689R		1.082	CHY	cytopl	conserved hypothetical protein
90	OE6278R		-1.242	NOF	cytopl	protein kinase afsK homolog
91	OE1117F		1.238	HY	cytopl	hypothetical protein
92	OE7045F	*tbpA*	-1.155	TC	cytopl	TATA-binding transcription initiation factor homolog
93	OE1196R		-1.081	HY	cytopl	hypothetical protein
94	OE2195F-2	*htr18*	-1.064	SIG	transm	transducer protein htr18
95	OE3108F	*pri*	1.095	RRR	cytopl	probable DNA primase
96	OE3646F	*nadB*	1.154	COM	anchN_lip	L-aspartate oxidase (EC 1.4.3.16) (quinolinate synthetase B, part of quinolinate synthetase complex)
97	OE5443F		1.073	REG	cytopl	probable transcription regulator
98	OE7012R		1.474	HY	cytopl	hypothetical protein
99	OE7222R		1.218	HY	cytopl	hypothetical protein
100	OE1817R		1.157	CHY	cytopl	conserved hypothetical protein
101	OE2019F	*fba1*	1.237	CIM	cytopl	fructose-bisphosphate aldolase (EC 4.1.2.13) 1
102	OE3125R		1.230	CHY	anchN_lip	conserved hypothetical protein
103	OE3611R	*basT*	-1.199	SIG	extrac	transducer protein basT
104	OE3936F	*pchB*	-1.170	NOF	cytopl	potassium channel protein homolog
105	OE4013R		1.120	HY	transm	hypothetical protein
106	OE4103R		-1.115	HY	cytopl	hypothetical protein
107	OE4569R		-1.080	HY	cytopl	hypothetical protein
108	OE4718F	*vacB*	1.183	NOF	cytopl	ribonuclease R homolog
109	OE7216F		1.165	NOF	cytopl	conserved hypothetical protein
110	OE2247R		-1.188	NOF	cytopl	conserved hypothetical protein
111	OE3284R		-1.318	NOF	cytopl	conserved hypothetical protein
112	OE1672F	*pyrE1*	1.126	NOF	cytopl	orotate phosphoribosyltransferase homolog
113	OE2120F		-1.114	NOF	cytopl	conserved hypothetical protein
114	OE3889R	*kefC*	-1.163	TP	transm	probable potassium transport protein kefC
115	OE3278R	*gcvT*	-1.115	AA	cytopl	glycine cleavage system protein T (aminomethyltransferase (EC 2.1.2.10))
116	OE1474R		1.284	HY	cytopl	hypothetical protein
117	OE1067R		1.267	CHY	cytopl	conserved hypothetical protein

Rank	ORF	Gene	Regulation (fold)	Function class[1]	Localization	Function of encoded protein
118	OE4722R	*hom*	-1.325	AA	cytopl	homoserine dehydrogenase (EC 1.1.1.3)
119	OE5162R-2	*orc5*	1.196	NOF	cytopl	cell division control protein cdc6 homolog
120	OE3923F	*lrp*	-1.143	REG	cytopl	global transcription regulator
121	OE1268F	*boa1*	-1.168	REG	cytopl	probable transcription regulator boa1
122	OE3206R		-1.094	CHY	cytopl	conserved hypothetical protein
123	OE1065R		1.199	CHY	cytopl	conserved hypothetical protein
124	OE1074F		1.130	ISH	cytopl	IS1341-type transposase (ISH12)
125	OE1121F		1.183	CHY	cytopl	conserved hypothetical protein
126	OE1229R		-1.078	HY	transm	hypothetical protein
127	OE1241R		-1.094	CHY	cytopl	conserved hypothetical protein
128	OE1272R	*mutS1*	-1.185	RRR	cytopl	DNA mismatch recognition protein
129	OE1353F		-1.189	HY	transm	hypothetical protein
130	OE1482R	*dpg*	1.115	CP	transm	probable dolichyl-phosphate beta-glucosyltransferase (EC 2.4.1.117) (only N-terminal homology)
131	OE1738R		-1.161	NOF	cytopl	hypothetical protein
132	OE2065R	*pepQ1*	-1.145	MIS	cytopl	probable X-Pro dipeptidase (EC 3.4.13.9)
133	OE2626R	*mrp*	1.135	MIS	cytopl	probable ATP-binding protein mrp
134	OE4612F	*hly*	-1.377	CP	extrac	halolysin R4 (EC 3.4.21.-)
135	OE4633F		-1.272	NOF	extrac	conserved hypothetical protein
136	OE5130F	*trkA4*	-1.112	NOF	cytopl	trkA domain protein
137	OE7024R	*gvpK1*	1.199	MIS	cytopl	gas-vesicle operon protein gvpK1
138	OE2367F	*aldH3*	1.083	MIS	cytopl	aldehyde dehydrogenase (glyceraldehyde-3-phosphate dehydrogenase homolog)
139	OE2225F	*dmsB*	1.311	EM	cytopl	dimethylsulfoxide reductase (EC 1.8.-.-) subunit B (electron transfer protein)
140	OE2372F	*acs3*	1.106	MIS	cytopl	acetate--CoA ligase (ADP-forming) (EC 6.2.1.13) (alpha and beta subunit fusion)
141	OE3479R		1.090	HY	cytopl	hypothetical protein
142	OE4054F		1.093	HY	cytopl	hypothetical protein
143	OE4398F	*hal*	-1.143	MIS	cytopl	O-acetylhomoserine (thiol)-lyase (EC 4.2.99.10)
144	OE4727R		1.265	ISH	cytopl	IS1341-type transposase (TCE31)
145	OE3036F	*glyA*	-1.164	AA	cytopl	glycine hydroxymethyltransferase (EC 2.1.2.1)
146	OE1399R	*tfbG*	-1.143	TC	cytopl	transcription initiation factor TFB
147	OE2049R		-1.081	NOF	cytopl	conserved hypothetical protein
148	OE3488R	*cre*	-1.212	AA	cytopl	probable creatininase (EC 3.5.2.10)
149	OE3671F		1.114	NOF	cytopl	conserved hypothetical protein
150	OE4544R		-1.172	CHY	cytopl	conserved hypothetical protein
151	OE3050F		1.167	NOF	cytopl	conserved hypothetical protein
152	OE3964R		-1.139	NOF	cytopl	conserved hypothetical protein
153	OE4741R	*rpoB1*	1.080	TC	cytopl	DNA-directed RNA polymerase (EC 2.7.7.6) subunit B'
154	OE1344R		1.165	HY	transm	hypothetical protein
155	OE3277R	*gcvH*	1.102	AA	cytopl	glycine cleavage system protein H
156	OE3087R		-1.215	CHY	transm	conserved hypothetical protein
157	OE1018F		1.153	MIS	transm	sugar transferase

Rank	ORF	Gene	Regulation (fold)	Function class[1]	Localization	Function of encoded protein
158	OE3131F		1.083	HY	cytopl	hypothetical protein
159	OE4028R		1.153	CHY	cytopl	conserved hypothetical protein
160	OE3162F		-1.106	NOF	cytopl	conserved hypothetical protein
161	OE3218F		-1.266	MIS	cytopl	cobalamin operon protein
162	OE7149F		-1.104	HY	cytopl	hypothetical protein
163	OE2220F		1.245	CHY	cytopl	conserved hypothetical protein
164	OE2622R	*porB*	1.235	CIM	cytopl	pyruvate--ferredoxin oxidoreductase (EC 1.2.7.1) beta subunit
165	OE3933F		1.142	HY	anchN_lip	hypothetical protein
166	OE4585R		-1.238	HY	transm	hypothetical protein
167	OE5022F		1.183	CHY	cytopl	conserved hypothetical protein
168	OE5202F	*pyrI*	1.249	NUM	cytopl	aspartate carbamoyltransferase (EC 2.1.3.2) regulatory subunit
169	OE6028R	*comA*	-1.086	CHY	anchN_lip	transforming DNA uptake protein homolog
170	OE2440F		-1.072	HY	transm	hypothetical protein
171	OE2758R		-1.092	CHY	cytopl	conserved hypothetical protein
172	OE2874F		-1.164	HY	cytopl	hypothetical protein
173	OE3118F		1.125	NOF	transm	conserved hypothetical protein
174	OE3392F	*rpl2*	-1.152	TL	cytopl	ribosomal protein L2
175	OE3595R	*moaD*	1.112	COM	cytopl	molybdopterin (MPT) converting factor, subunit 1
176	OE4146F	*tbpE*	1.290	TC	cytopl	TATA-binding transcription initiation factor
177	OE4468F		-1.138	CHY	cytopl	conserved hypothetical protein
178	OE5127F	*gvpC2*	-1.269	CP	cytopl	gas-vesicle protein gvpC2
179	OE5187R		-1.225	MIS	extrac	probable hydrolase
180	OE6340R		1.124	CHY	cytopl	conserved hypothetical protein
181	OE7003R		1.185	CHY	cytopl	conserved hypothetical protein
182	OE4022R	*manC*	-1.255	CHM	cytopl	mannose-1-phosphate guanylyltransferase (EC 2.7.7.13)
183	OE5186R	*perA*	-1.189	MIS	cytopl	catalase (EC 1.11.1.6) (including: peroxidase (EC 1.11.1.7))
184	OE3629R		1.090	TP	anchN_lip	ABC-type transport system periplasmic substrate-binding protein
185	OE1549F		1.104	NOF	cytopl	conserved hypothetical protein
186	OE2577R		1.244	NOF	cytopl	conserved hypothetical protein
187	OE3204R		-1.127	CHY	cytopl	conserved hypothetical protein
188	OE3330F		-1.126	CHY	transm	conserved hypothetical protein
189	OE4683F	*ribC, risA*	-1.183	COM	cytopl	riboflavin synthase (EC 2.5.1.9) alpha subunit
190	OE1853R		1.110	CHY	transm	Na+/cholate efflux system protein mrpF homolog
191	OE4613F	*acn*	1.147	CIM	cytopl	aconitate hydratase (EC 4.2.1.3)
192	OE1016R	*graD2*	-1.170	CHM	cytopl	glucose-1-phosphate thymidylyltransferase (EC 2.7.7.24)
193	OE1887F		-1.172	HY	transm	hypothetical protein
194	OE3927F		-1.101	CHY	transm	conserved hypothetical protein
195	OE1357F		-1.147	HY	transm	hypothetical protein
196	OE1867R		1.271	HY	transm	hypothetical protein
197	OE3949R		1.259	CHY	cytopl	glutaredoxin homolog

Rank	ORF	Gene	Regulation (fold)	Function class[1]	Localization	Function of encoded protein
198	OE7065F	*cydA1*	-1.233	EM	transm	cytochrome d ubiquinol oxidase (EC 1.10.3.-) subunit I
199	OE7139R		1.077	HY	cytopl	hypothetical protein
200	OE1197R		1.087	HY	cytopl	hypothetical protein
201	OE3143R		-1.420	CP	cytopl	tetrahedral aminopeptidase
202	OE4018F		-1.120	CHY	cytopl	conserved hypothetical protein
203	OE4607R	*flaG2*	1.076	CHY	extrac	flaG protein homolog
204	OE7176R		1.130	CHY	cytopl	helicase homolog
205	OE1633F		-1.127	CHY	cytopl	conserved hypothetical protein
206	OE3195F	*sucB*	1.078	CIM	cytopl	succinate--CoA ligase (ADP-forming) (EC 6.2.1.5) beta subunit
207	OE3749R	*tgtA1*	1.132	RMT	cytopl	queuine tRNA-ribosyltransferase (EC 2.4.2.29)
208	OE1711R	*korA*	1.180	CIM	cytopl	oxoglutarate--ferredoxin oxidoreductase (EC 1.2.7.3) alpha subunit
209	OE2054F		-1.172	CHY	cytopl	conserved hypothetical protein
210	OE2633F	*rpl13*	1.201	TL	cytopl	ribosomal protein L13
211	OE3319R	*cbiM*	1.172	TP	transm	CbiM protein (unassigned function) (probable ABC-type cobalt transport system permease protein 2)
212	OE4753R		-1.144	NOF	cytopl	transcription regulator homolog
213	OE1956F	*nuoCD*	1.267	EM	cytopl	NADH dehydrogenase (ubiquinone) (EC 1.6.5.3) subunit CD
214	OE4355R	*tssA*	1.159	NOF	cytopl	thiosulfate sulfurtransferase homolog
215	OE3782R		-1.215	CHY	transm	conserved hypothetical protein
216	OE2370R	*gufA*	-1.174	MIS	transm	gufA protein
217	OE2734F	*hutU*	1.204	AA	cytopl	urocanate hydratase (EC 4.2.1.49)
218	OE1171F	*rmeMa*	1.155	MIS	cytopl	type I restriction-modification system DNA-methyltransferase rmeM (nonfunctional, N-terminal part)
219	OE4644R		-1.129	NOF	cytopl	conserved hypothetical protein
220	OE2138F	*acd2*	-1.185	LIP	cytopl	probable acyl/butyryl-CoA dehydrogenase (EC 1.3.99.-)
221	OE3136F		-1.212	CHY	cytopl	conserved hypothetical protein
222	OE3829R		1.122	CHY	cytopl	conserved hypothetical protein
223	OE4118R	*leuS*	1.130	TL	cytopl	leucine--tRNA ligase (EC 6.1.1.4)
224	OE1120F		1.192	CHY	cytopl	conserved hypothetical protein
225	OE4165R	*pcn*	1.178	NOF	cytopl	DNA-directed DNA polymerase sliding clamp homolog
226	OE1874R	*petB*	1.198	EM	transm	probable menaquinol--cytochrome-c reductase (EC 1.10.2.-) (cytochrome bc complex) cytochrome b subunit
227	OE5184F		-1.138	CHY	transm	conserved hypothetical protein
228	OE7027R	*gvpI1*	1.266	MIS	cytopl	gas-vesicle operon protein gvpI1
229	OE1025F		-1.099	CHY	cytopl	conserved hypothetical protein
230	OE1490R		-1.131	NOF	cytopl	conserved hypothetical protein
231	OE3347F	*htrI*	-1.106	SIG	cytopl	transducer protein htrI
232	OE4354R		1.123	CHY	cytopl	conserved hypothetical protein
233	OE3376F	*crtB2*	1.137	LIP	cytopl	geranylgeranyl-diphosphate geranylgeranyltransferase (EC 2.5.1.32) (phytoene synthase)
234	OE2512R	*hpcE*	1.060	CHY	cytopl	2-hydroxyhepta-2,4-diene-1,7-dioate isomerase homolog
235	OE3273F	*tfx*	-1.135	MIS	cytopl	DNA-binding protein
236	OE4047R		1.153	MIS	cytopl	phage PhiH1 repressor protein homolog
237	OE4555F	*dppC1*	-1.222	TP	transm	ABC-type transport system permease protein

Rank	ORF	Gene	Regulation (fold)	Function class[1]	Localization	Function of encoded protein
238	OE5118R	*gvpI2*	-1.395	MIS	cytopl	gas-vesicle operon protein gvpI2
239	OE3045F		-1.256	NOF	cytopl	conserved hypothetical protein

(1) see www.halolex.mpg.de for further explanation
